# Efficient Path Planning and Truthful Incentive Mechanism Design for Mobile Crowdsensing

**DOI:** 10.3390/s18124408

**Published:** 2018-12-13

**Authors:** Xi Tao, Wei Song

**Affiliations:** Faculty of Computer Science, University of New Brunswick, Fredericton, NB E3B 5A3, Canada; xtao@unb.ca

**Keywords:** mobile crowdsensing, path planning, mechanism design, truthfulness

## Abstract

Mobile crowdsensing (MCS) is a promising paradigm for large-scale sensing. A group of users are recruited as workers to accomplish various sensing tasks and provide data to the platform and requesters. A key problem in MCS is to design the incentive mechanism, which can attract enough workers to participate in sensing activities and maintain the truthfulness. As the main advantage of MCS, user mobility is a factor that must be considered. We make an attempt to build a technical framework for MCS, which is associated with a truthful incentive mechanism taking the movements of numerous workers into account. Our proposed framework contains two challenging problems: path planning and incentive mechanism design. In the path planning problem, every worker independently plans a tour to carry out the posted tasks according to its own strategy. A heuristic algorithm is proposed for the path planning problem, which is compared with two baseline algorithms and the optimal solution. In the incentive mechanism design, the platform develops a truthful mechanism to select the winners and determine their payments. The proposed mechanism is proved to be computationally efficient, individually rational, and truthful. In order to evaluate the performance of our proposed mechanism, the well-known Vickrey–Clarke–Groves (VCG) mechanism is considered as a baseline. Simulations are conducted to evaluate the performance of our proposed framework. The results show that the proposed heuristic algorithm for the path planning problem outperforms the baseline algorithms and approaches the optimal solution. Meanwhile, the proposed mechanism holds a smaller total payment compared with the VCG mechanism when both mechanisms achieve the same performance. Finally, the utility of a selected winner shows the truthfulness of proposed mechanism by changing its bid.

## 1. Introduction

Mobile crowdsensing (MCS) is an emerging technique to leverage the capacities of mobile devices (e.g., smartphones, tablet computers, and wearables) in large-scale sensing and computing. The term “mobile crowdsensing” was first coined by Ganti in [1]. Thanks to the explosive growth of mobile devices, MCS has attracted more and more attention in recent years [2,3]. MCS enables a different way to sense based on the great quantity of mobile devices, which has several advantages over the traditional sensing methods. For example, MCS is able to cover a large sensing area without deploying a wireless sensor network, since a mass of users can be recruited from a huge user pool to satisfy the sensing requirements. These advantages make MCS suitable for a broad range of sensing applications, e.g., environment monitoring [4], traffic management [5], and healthcare [6]. Waze [7] is a typical representative of MCS applications, which receives the traffic reports from smartphone users to provide navigation information and route details back to mobile users.

Although MCS possesses so many advantages, there still exist some challenges to implement an MCS framework. One key problem is to design an appropriate incentive mechanism, which is the component to attract mobile users as workers to participate in the sensing activities and maintain a sufficient number of workers in the framework [8,9,10]. Basically, there are two main functions of the incentive mechanism. The first one is the method to select workers from the user pool taking some specific requirements into account. For example, the mechanism needs to select workers who can provide high-quality sensing data when the data quality is the target of the framework, while spatially dispersed users can be hired when the target is the coverage. The second function of the mechanism is to determine the payments after recruiting the workers. It is well known that the budget is a critical restriction on a project. Thus, the requesters generally set a budget after posting their tasks on the framework. In this case, it is desirable to use a method that spends the budget wisely to recruit a group of workers.

Moreover, user mobility offers the benefit of allowing workers to travel around and finish tasks, but also raises an important question on who should be the decision maker for the workers’ paths [11,12]. On one hand, the platform is considered as the path planner, since it has more computing power and information to obtain a global optimal solution. The platform is able to coordinate all workers when it assigns the tasks to the workers. This is termed the platform-centric mode. The drawback of this mode is that the platform has to collect all the information of workers, e.g., the locations and moving trajectories. The information collection costs substantial efforts and may also infringe on the privacy of workers. On the other hand, the workers can decide their paths on their own based on individual strategies in order to improve their autonomy. This is termed the worker-centric mode. In this mode, the workers just provide the data samples to the platform without revealing their location information, which is a great benefit for the privacy of workers. The worker-centric mode also has some weak points. For example, without the central coordination or awareness of each other’s choice, potential competition may exist among the workers when they contend for the same tasks.

**Figure 1:**
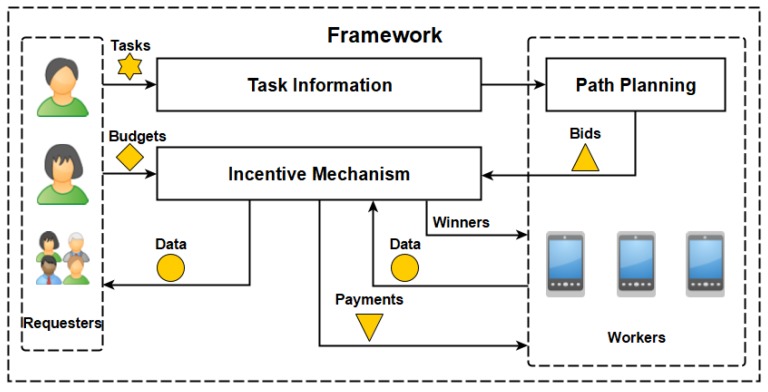
The proposed technical framework for mobile crowdsensing.

In this paper, we aim at building a technical framework for MCS that involves an incentive mechanism and takes user mobility into account. The main structure of our proposed framework is shown in Figure 1. First, we propose a location-protected method to assign the tasks to the workers by leveraging the worker-centric mode. The workers plan their paths to accomplish the tasks according to their own strategies. Then, the workers submit their bids to the platform without revealing any location information, which is called location-protected. A heuristic bidirectional searching algorithm is proposed to solve the path planning problem for the workers. Second, the platform conducts an auction after receiving the bids from the workers to determine the winners and payments. A truthful incentive mechanism is designed to select winners from the bidders according to the assignment rule and determine the payments by a payment rule. Finally, the selected winners submit their collected data samples to the platform to receive the reward payments.

Specifically, our main contributions are three-fold.

- We build an MCS framework including two phases, path planning and incentive mechanism design. The separation between the path planning and the incentive mechanism design makes the proposed framework balanced since neither the platform (or requesters) nor the workers play a dominant role. The workers can choose to participate in the sensing activities or not during the phase of path planning, while the platform has the authority to select the workers as winners and pay the winners. Therefore, the proposed framework gives all stakeholders a chance to develop their own strategies and optimize their utilities.
- In the phase of path planning, a heuristic bidirectional searching algorithm is proposed to plan the paths of workers. The proposed heuristic algorithm can leverage the current remaining resources to explore a possibility in the near future, which improves the performance of the searching. Compared with two baseline algorithms and the optimal solution, our proposed heuristic algorithm outperforms the baseline algorithms and approaches the optimal solution.
- In the phase of incentive mechanism design, a truthful mechanism is designed for the platform, which is proved to be computationally efficient, individually rational, and truthful. It is worth pointing out that the proposed mechanism is truthful not only in the task sets of workers but also in the cost bids of workers. To evaluate the proposed mechanism, the well-known Vickrey–Clarke–Groves (VCG) mechanism is considered as a baseline and the results show that the proposed mechanism spends a smaller total payment than the VCG mechanism with the same performance.

The remainder of this paper is organized as follows. The related work is presented in Section 2. Section 3 gives the system model and problem formulation. We present the details of the proposed solutions in Section 4. The simulation results are shown in Section 5. Section 6 concludes this paper.

## 2. Related Work and Technical Background

In this section, we review the related work on MCS. For ease of comparison, we classify the existing solutions into two groups: i.e., incentive mechanism design and task allocation.

### 2.1. Incentive Mechanism Design

As already known, the incentive mechanism design is an important and also challenging problem in MCS. In some survey papers, a variety of techniques are reviewed for designing the incentive mechanism [8,9,10,13,14]. The most widely used method is auction theory. Auction theory is an effective tool to design the incentive mechanism, in which bidders submit their bids to the auctioneer; then, the auctioneer selects the winners and determines the payments [15]. Applying auction theory to design the incentive mechanism for mobile crowdsensing, researchers regard the platform as the auctioneer and the workers as the bidders. The platform designs and announces the assignment rule and the payment rule to the workers, while the workers submit their bids to compete for the tasks. In general, there are three desired properties when implementing an incentive mechanism by auction theory, i.e., computational efficiency, individual rationality, and truthfulness. The computational efficiency requires the mechanism to obtain the results within a polynomial time complexity. The individual rationality means that the utilities of bidders cannot be negative with the auction outcome. The mechanism is truthful if the bidders maximize their utilities by bidding their true costs or values. As already seen, these three properties are essential to ensure implementability of the incentive mechanism in practice. Meanwhile, these properties also pose challenges to the design.

Although auctions are easy to implement and operate, it can be hard to ensure the truthfulness of the mechanism, especially when the allocation problem is computationally intractable. For example, though VCG auction is a general method to design truthful mechanisms, the truthfulness of the VCG auction rules is lost if the optimal outcome is not available [16,17]. As a result, mechanism designers need to find a different way to achieve truthfulness when the allocation problem is hard to solve. In addition, there is another problem with using auctions. It is essential to maintain a large group of workers, which is the foundation of MCS. Since auctions are strongly competitive, “weak” workers do not even have a chance to win and they would opt out of the auctions in the future [18]. Therefore, other methods are also proposed in incentive mechanism design, e.g., lottery [13] and bargaining [19].

Yang et al. [20,21] considered the incentive mechanism design in both the platform-centric model and user-centric model. In the platform-centric model, a Stackelberg game is used as the incentive mechanism. The platform as the leader in the game announces its total reward at the beginning. Then, the workers are motivated to participate in the sensing based on their strategies and the announced reward. In the worker-centric model, an auction-based incentive mechanism is proposed, which is proved to be computationally efficient, individually rational, profitable, and truthful. The authors firstly designed a reverse auction based on the Local Search-Based (LSB) auction to achieve an approximation of the optimal solution. However, the LSB auction cannot ensure truthfulness. In order to achieve truthfulness, a novel auction mechanism called MSensing is proposed by leveraging the well-known Myerson’s theorem [22]. The simulation results show that truthfulness is guaranteed in the proposed mechanism.

In [20], the bids are the sensing time of workers. Differently, Wang et al. [23] presented an incentive mechanism taking the quality of crowd (QoC) into account. The QoC is the measurement of the workers’ potential capacities to provide high-quality data or services. Four different models are defined to calculate the value of QoC, i.e., the linear model, probabilistic coverage model, logarithmic model, and hyperbolic tangent model. The proposed mechanism takes the QoC and costs of workers as bids and selects winners to minimize the total cost while satisfying a quality requirement. The truthfulness of the mechanism is proved by leveraging Myerson’s theorem. The simulation results show that the proposed incentive mechanism produces the near-optimal solutions.

In addition to truthfulness, how to maintain a large user group is another key problem in the long-term sensing. Lee et al. [24,25] designed a reverse auction based dynamic price (RADP) mechanism to maintain an adequate number of workers. The proposed mechanism prevents the workers from dropping out of sensing activities by virtual participant credit (VPC), which is provided to workers who lose in the previous auction. The VPC plays two roles during the long-term sensing. First, it improves the winning probability of weak workers if the workers keep losing all the time. Second, it would compensate the workers for staying in the auction by an extra credit when the workers win the auction. As a result of winning, the VPC is paid to the winners at once and then reset to zero. In addition, the mechanism reveals the highest selling price to the dropped workers to attract them back to the auction. Compared with a random-selection-fixed-price incentive mechanism, the simulation results show that the proposed mechanism not only decreases the cost but also maintains the desired number of workers.

We have reviewed two key challenges in incentive mechanism design, i.e., truthfulness and long-term sensing. Apparently, there are many other research directions. For instance, if the cost budget is changed to the payment budget, the incentive mechanism design would target a budget feasible mechanism [26,27]. Instead of truthfulness, some efforts are made to solve the intractable allocation problem by using a randomized mechanism to achieve truthfulness in expectation [28,29]. In the long-term sensing, online mechanisms have started to attract researchers’ attention. Several works have been published in the area of online mechanism design [30,31]. In addition, privacy is another focus in recent research on incentive mechanism design [32,33].

The research so far mainly focuses on the single-parameter environment, which can leverage Myerson’s theorem to prove the truthfulness. In some studies, the workers just need to bid their costs on the platform and their task sets are assumed to be truthfully submitted to the platform. However, in the worker-centric mode, which is considered in our work, the workers may manipulate their task sets because they are allowed to plan their paths. As a result, the platform cannot be assumed to know a priori the tasks to be completed by each worker. The workers should bid both their task sets and the corresponding costs to the platform. Myerson’s theorem cannot be applied in this situation, since it is not a single-parameter environment any more. In addition, the VCG mechanism is also not viable due to the computational intractability of the winner selection problem. To address these issues, we aim to design an incentive mechanism that is computationally efficient and truthful with respect to both the task sets and costs of workers.

### 2.2. Task Allocation

Task allocation aims to efficiently assign the tasks among the workers. It is a key problem of MCS and directly determines the performance of an MCS application. An efficient task allocation method can reduce the costs of the workers and further save the budget of the platform and requesters. The task allocation problem has been addressed in different perspectives, e.g., with single task or multiple tasks, and with the goals of low cost or quality enhancement [34]. In our proposed framework, we intend to address the task allocation problem by taking user mobility into account and thereby turning it into the path planning problem.

Similar to our path planning problem, He et al. [35,36] considered the mobility of workers and proposed a task allocation problem within the travelling distance limit. The target of the platform is to maximize the value of tasks by properly recruiting workers who collect the data samples. In order to suppress data redundancy, the platform limits the maximum number of workers for each task. A local-ratio-based algorithm is proposed to achieve the target of the platform, which is shown to be an efficient solution by the simulation results.

The data quality and data cost are two fundamental elements in task allocation [37]. There is a trade-off between the data quality and the data cost. Improving the data quality requires the platform to spend more to attract the workers who are able to provide higher data quality. That is, the improvement of data quality may cause an increase of the data cost. Zhou et al. [38] and Zhang et al. [39] both considered the data quality and data cost. However, the data quality and data cost have different definitions. In [38], the authors aimed at maximizing the data quality at a cost of the travelling distance, while, in [39], the data quality is defined as the coverage and the target of the platform is to maximize the coverage of the tasks under the budget constraint.

Some previous works formulate the task allocation problem as the set cover problem, in which each worker has a sensing range and can complete one or more tasks located within the range. Then, the platform assigns the tasks to the workers to achieve a maximum coverage of the tasks as in the platform-centric mode. Here, a task is covered when it is finished by at least one worker. However, user mobility is often neglected in such formulation. In our study, we formulate the task allocation problem as the path planning problem in the worker-centric mode, taking user mobility into account. Compared with the previous studies for the platform-centric mode, our formulation allows the workers to be more influential and more independent.

## 3. System Model and Problem Formulations

In this section, we first provide the system model for our proposed technical framework. Then, we formulate the path planning problem and the incentive mechanism design problem in detail. Finally, we analyze the computational hardness of our formulated problems. For easy reference, we list the important notations used in this paper in Table 1.

### 3.1. System Model

As shown in Figure 1, the requesters post their tasks to the platform and each worker plans the path based on the posted task information. Then, the incentive mechanism receives the budgets from the requesters and the bids from the workers. After the winner selection and payment determination, the incentive mechanism returns the data that are provided by the winners to the requesters and the payments to the workers. Therefore, there are two basic problems in our proposed framework, i.e., the path planning problem for workers and the incentive mechanism design for the platform.

**Figure 2:**
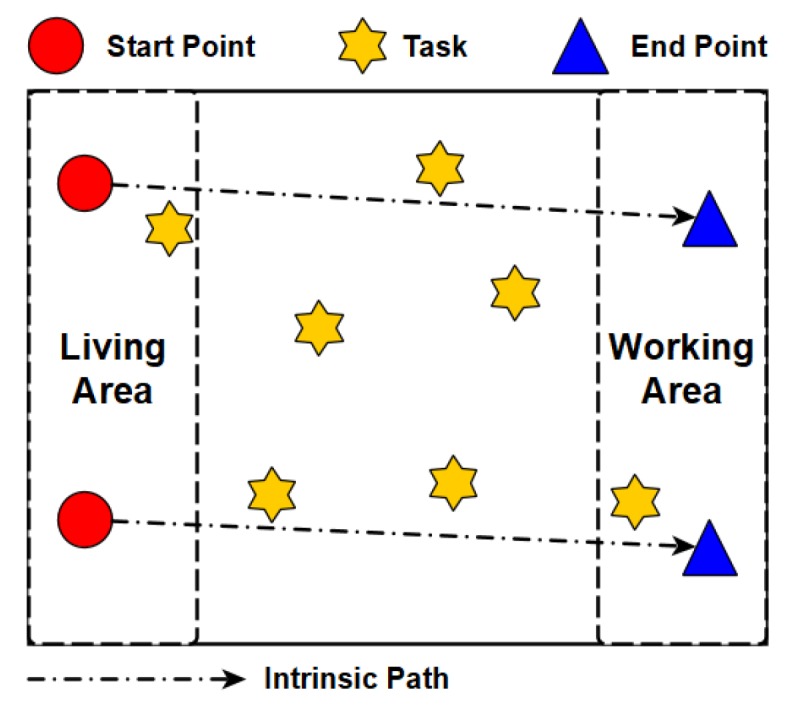
An example scenario.

When it comes to the path planning, we consider a special scenario shown in Figure 2. There are a set of tasks and a set of workers, denoted by $T=\{t_{1},t_{2},\ldots ,t_{j},\ldots ,t_{m}\}$ and $W=\{w_{1},w_{2},\ldots ,w_{i},\ldots ,w_{n}\}$, respectively. The tasks are uniformly distributed in the area, while the workers often move from the start points (e.g., houses) to the end points (e.g., working places). The start points and end points of workers follow two different uniform distributions. As shown in Figure 2, the direct paths from the start points to the end points are called the intrinsic paths of workers. In addition, for every posted task $t_{j}\in T$, its value and energy cost are defined by its requester, which are denoted by $v_{j}$ and $g_{j}$, respectively. Note that our proposed solutions are not limited by this sensing scenario.

### 3.2. Path Planning

Since the workers are given the autonomy to plan their own paths, the workers desire to find a strategy to improve their competitiveness. Without loss of generality, we assume that the platform makes the decision on behalf of requesters in the following. It is known to the workers that the requesters or the platform intend to achieve the largest value of the posted tasks. Therefore, it is a natural strategy for the workers to choose tasks of large value. However, the resources of workers are limited. The workers have to plan their paths under some constraints. First of all, every worker $w_{i}\in W$ has the fixed start point and end point, which leads to an intrinsic path with length $h_{i}$. Second, for every worker $w_{i}\in W$, there is an energy limit $\theta_{i}$, which is the maximum battery power that worker $w_{i}$ is willing to spend on sensing. At last, every worker $w_{i}\in W$ has a maximum travelling distance $l_{i}$, which is larger than the length $h_{i}$ of the intrinsic path. Since every worker is an independent planner, there are *n* path planning problems and the problem for worker $w_{i}$ is formulated as follows:

(1a) $$\begin{array}{c} \max.\;\;\;\sum_{t_{j}\in F_{i}}v_{j}, \end{array}$$

(1b) $$\begin{array}{c} s.t.\;\;\;\;\;d\left(F_{i}\right)\leq l_{i}, \end{array}$$

(1c) $$\begin{array}{c} \;\;\;\;\;\;\;\;\sum_{t_{j}\in F_{i}}g_{j}\leq \theta_{i}, \end{array}$$

where $F_{i}$ denotes the designed path of worker $w_{i}$ and $d(F_{i})$ is a function to calculate the length of path $F_{i}$.

### 3.3. Incentive Mechanism Design

The platform collects the budgets from the requesters and the bids from the workers. Here, we assume that the platform sets a budget *B* for the total cost of tasks after integrating all the budgets of the requesters. For each worker $w_{i}\in W$, the bid is a pair of the task set and the cost, which is denoted by $\left(S_{i},b_{i}\right)$. Since the true task set of worker $w_{i}$ is the set of tasks along path $F_{i}$, for notation convenience, we also use $F_{i}$ to represent the true task set of worker $w_{i}$. As seen here, worker $w_{i}$ only submits the bid to the platform without any location information to protect the privacy. The true cost of worker $w_{i}$ is defined to be proportional to the additional travelling distance beyond that of the intrinsic path, which is the difference between total travelling distance $d(F_{i})$ and the length $h_{i}$ of the intrinsic path. Specifically, the true cost $c_{i}$ of worker $w_{i}$ is characterized by

(2) $$\begin{array}{c} c_{i}\left(F_{i}\right)=\gamma_{i}\times \left(d\left(F_{i}\right)-h_{i}\right), \end{array}$$

where $\gamma_{i}$ is the true cost per distance of worker $w_{i}$.

The true cost in Equation (2) depends on the real expenses of the work for travelling the additional distance. However, worker $w_{i}$ may not submit its true task set along path $F_{i}$ to improve its winning chance. For example, worker $w_{i}$ may include task $t_{j}\notin F_{j}$ in its bid $S_{i}$. Task $t_{j}$ may be to take a photo of a building. Suppose that worker $w_{i}$ wins, but it will not pass by the building when travelling along path $F_{i}$ and thus cannot complete this task. Thus, worker $w_{i}$ just submits a dog’s photo pretending to have finished the task. In this case, the platform can catch that worker $w_{i}$ is cheating using many techniques to detect such fake data, e.g., machine learning methods. Then, worker $w_{i}$ is deprived of the payment and even subject to more serious punishment. As seen, such cheating behaviour can cause a prohibitively large cost to the worker. Accordingly, we model these characteristics of the cost bid $b_{i}$ of worker $w_{i}$ for set $S_{i}$ as follows:

(3) $$\begin{array}{c} b\left(S_{i}\right)=\left\{\begin{array}{cc} b_{i}, & S_{i}\subseteq F_{i},\\ +\infty , & S_{i}⊈F_{i}. \end{array}\right. \end{array}$$

Here, if worker $w_{i}$ bids fewer tasks in the task set, i.e., $S_{i}\subseteq F_{i}$, the cost bid is still $b_{i}$ since the platform cannot find any fake data in the bid of worker $w_{i}$. On the other hand, if worker $w_{i}$ bids for some tasks that are not included in its path $F_{i}$, i.e., $S_{i}⊈F_{i}$, the cost to worker $w_{i}$ can be infinitely large. That is, the platform can punish worker $w_{i}$ and exclude it from this and any future bidding.

Next, we define the task value in more detail. Although every task has its own value, it is still a problem for the platform to evaluate the total value of a task when duplicate data samples are received. This is known as the data redundancy problem. With data redundancy, it is possible for the platform to spend too much budget on a specific task, which is destructive to the coverage of tasks. To solve the data redundancy problem, we employ the cumulative value of a task by taking into account all competitors for it. If we denote the assignment of winners by $\left\{x_{i}|\forall w_{i}\in W\right\}$, where $x_{i}=1$ indicates worker $w_{i}$ is selected as a winner and otherwise $x_{i}=0$. Then, for every task $t_{j}\in T$, the number of competitors for it, denoted by $y_{j}$, is calculated by

(4) $$\begin{array}{c} y_{j}=\sum_{w_{i}\in W}x_{i}\cdot 1\left(t_{j}\in S_{i}\right), \end{array}$$

where 1(·) is the indicator function, which equals 1 when the condition in the function argument is satisfied. For every task $t_{j}\in T$, its original value is $v_{j}$. Then, its cumulative value, denoted by ${\hat{v}}_{j}$, follows the diminishing marginal increase and is characterized by

(5) $$\begin{array}{c} {\hat{v}}_{j}=v_{j}\times \log_{2}\left(1+y_{j}\right). \end{array}$$

Since the true task set and the true cost are the private information of worker $w_{i}$, the bids received by the platform may not be the true information. As a result, the platform has to design an incentive mechanism to motivate the workers to reveal their true information. The incentive mechanism needs to solve two problems, i.e., the winner selection and payment determination. The result of winner selection is the foundation to determine the payments. Thus, we consider the winner selection problem first.

Based on the above definitions for costs of workers and values of tasks, the winner selection problem is formulated as follows:

(6a) $$\begin{array}{c} \max.\;\;\;\sum_{t_{j}\in T}{\hat{v}}_{j}, \end{array}$$

(6b) $$\begin{array}{c} s.t.\;\;\;\;\;\sum_{w_{i}\in W}x_{i}\cdot b\left(S_{i}\right)\leq B, \end{array}$$

(6c) $$\begin{array}{c} \;\;\;\;\;\;\;\;x_{i}=\left\{0,1\right\},\forall w_{i}\in W. \end{array}$$

In problem (6), the objective of the platform is to maximize the cumulative value of the completed tasks. In addition, the total cost of all winners cannot be larger than the budget of total cost. After the winner selection, a payment rule needs to be applied to determine the payments to the winners, which makes the incentive mechanism complete.

### 3.4. Computational Hardness

In the following, we first analyze the computational hardness of the path planning problem.

**Theorem** **1.** *The path planning problem in Equation (1) is NP-hard.*

**Proof.**  First, we consider the decision form of problem (1), given by

(7a) $$\begin{array}{c} \mathrm{find}.\;\;\;F_{i},\mathrm{with}\sum_{t_{j}\in F_{i}}v_{j}\geq \rho , \end{array}$$

(7b) $$\begin{array}{c} s.t.\;\;\;\;\;d\left(F_{i}\right)\leq l_{i}, \end{array}$$

(7c) $$\begin{array}{c} \;\;\;\;\;\;\;\;\sum_{t_{j}\in F_{i}}g_{j}\leq \theta_{i}. \end{array}$$

As seen, the decision form is to obtain a yes-or-no answer to the problem that whether the given path $F_{i}$ has a value not less than the threshold ρ. Obviously, the decision form in Equation (7) is NP, since it only takes a polynomial time at most of O(m) to verify the constraints and compare the objective value with the threshold ρ.

Next, we prove that problem (7) is NP-complete, by reducing a known NP-complete problem, i.e., the decision form of the knapsack problem, to an instance of problem (7). The knapsack problem is to select a subset from a given set of items, each item with a weight and a value, to maximize the total value under a limit of the total weight. The decision form of the knapsack problem is to determine whether a value of ν can be achieved without exceeding the weight ω.

In the following, we construct an instance of problem (7) to solve the decision form of the knapsack problem. In constraint (7b), we assume that there is a very large maximum travelling distance $l_{i}$ of worker $w_{i}$, which is enough to take all the tasks. As a result, we only need to consider the energy constraint (7c). The energy costs of tasks correspond to the weights of items in the knapsack problem and the energy limit $\theta_{i}$ is mapped to the total weight ω. The values of tasks and the total value are mapped to the values of items and the total value ν in the knapsack problem, respectively. Therefore, our constructed instance of problem (7) is exactly a decision form of the knapsack problem, which is known to be NP-complete. Thus, the decision form of the path planning problem (7) is also NP-complete. Hence, the corresponding optimization form defined in Equation (1) is NP-hard. □

As proved in Theorem 1, the path planning problem is NP-hard. However, when the problem size is small, we can reformulate it as an integer linear program (ILP). Since the path planning problem is essentially to find a path that traverses a subset of tasks under certain constraints, we reformulate it as a graph-based ILP in the following:

(8a) $$\begin{array}{c} \max.\;\;\;\sum_{k=1}^{m}v_{k}\left(\sum_{j=0}^{m+1}q_{j,k}\right), \end{array}$$

(8b) $$\begin{array}{c} s.t.\;\;\;\;\;\sum_{j=0}^{m+1}\sum_{k=0}^{m+1}d_{j,k}\cdot q_{j,k}\leq l_{i}, \end{array}$$

(8c) $$\begin{array}{c} \;\;\;\;\;\;\;\;\;\sum_{k=1}^{m}g_{k}\left(\sum_{j=0}^{m+1}q_{j,k}\right)\leq \theta_{i}, \end{array}$$

(8d) $$\begin{array}{c} \;\;\;\;\;\;\;\;\sum_{j=0}^{m+1}q_{j,k}\leq 1,\forall k\in \left[0,m+1\right], \end{array}$$

(8e) $$\begin{array}{c} \;\;\;\;\;\;\;\;\sum_{k=0}^{m+1}q_{j,k}\leq 1,\forall j\in \left[0,m+1\right], \end{array}$$

(8f) $$\begin{array}{c} \;\;\;\;\;\;\;\;\sum_{k=0}^{m+1}\left(q_{j,k}-q_{k,j}\right)=\left\{\begin{array}{cc} 1, & j=0,\\ 0, & \forall j\in [1,m],\\ -1, & j=m+1, \end{array}\right. \end{array}$$

(8g) $$\begin{array}{c} \;\;\;\;\;\;\;\;r_{j,k}\leq \left(m+1\right)\cdot q_{j,k},\forall j\in \left[0,m+1\right],\forall k\in \left[0,m+1\right], \end{array}$$

(8h) $$\begin{array}{c} \;\;\;\;\;\;\;\;\sum_{k=0}^{m+1}r_{0,k}=\sum_{j=0}^{m+1}\sum_{k=1}^{m+1}q_{j,k}, \end{array}$$

(8i) $$\begin{array}{c} \;\;\;\;\;\;\;\;q_{j,j}=0,\forall j\in \left[0,m+1\right], \end{array}$$

(8j) $$\begin{array}{c} \;\;\;\;\;\;\;\;\sum_{j=0}^{m+1}r_{j,k}-\sum_{j=0}^{m+1}r_{k,j}=\sum_{j=0}^{m+1}q_{j,k},\forall k\in \left[1,m\right], \end{array}$$

(8k) $$\begin{array}{c} \;\;\;\;\;\;\;\;q_{j,k}=\left\{0,1\right\},\forall j\in \left[0,m+1\right],\forall k\in \left[0,m+1\right], \end{array}$$

(8l) $$\begin{array}{c} \;\;\;\;\;\;\;\;r_{j,k}=\left\{0,1,\ldots ,m+1\right\},\forall j\in \left[0,m+1\right],\forall k\in \left[0,m+1\right]. \end{array}$$

Here, for every worker $w_{i}$, there are (m+2) nodes in the path planning problem including the start point of worker $w_{i}$, *m* tasks, and the end point of worker $w_{i}$. Then, we can construct a weighted bidirectional graph with these (m+2) nodes, in which the starting point only has outgoing edges to all other nodes, the end point only has incoming edges, and, for the remaining *m* nodes, each has an edge to the other nodes. The weight for an edge between two nodes is the travelling distance between them. Based on this graph, the path planning problem is to find a path, represented by $q_{j,k},\forall j,\forall k$, such that the distance and energy cost limits are satisfied. Here, $q_{j,k}$ equals 1 if the path solution includes the edge from node *j* to node *k* and $q_{j,k}$ equals 0 otherwise.

**Figure 3:**
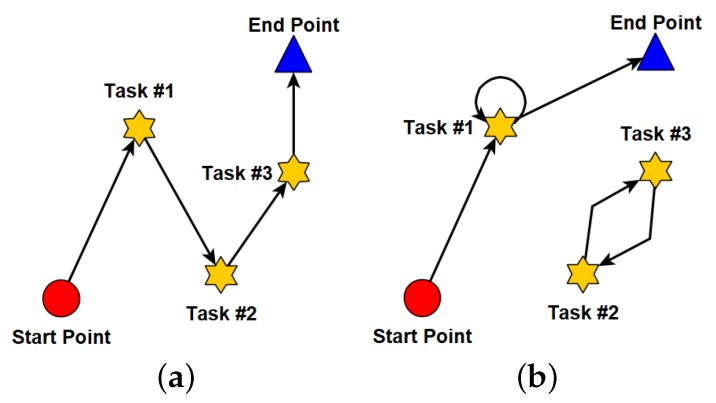
Paths without loops and with loops. (**a**) without loops; (**b**) with loops.

Based on the graph model and the path representation, Equation (8a) is the objective function to maximize the value of the path. Equation (8b) is the travelling distance limit and Equation (8c) is the constraint of energy cost. Equations (8d–f) define the in-degree, out-degree, and their relationship with each node. As seen, Equations (8d–f) ensure that the solution is a path, in which the degree of each node on the path is restricted by 1, and the start and end points of worker $w_{i}$ are the first and last nodes of the path, shown in Figure 3a.

To ensure that the path solution as shown in Figure 3a does not include any loop, we use additional variables $r_{j,k}$, which can be understood as some flow amount for the edge from node *j* to node *k*. Consider that the start point of worker $w_{i}$ produces a flow of an amount at most (m+1), and each other node consumes one unit of the flow. Then, the variables $r_{j,k}$ can be used to ensure that the path defined by $q_{j,k}$ is fully connected and loop-free. First, constraint (8g) means that, if the path does not include an edge between two nodes, there is no flow between them. Then, constraint (8h) defines the initial flow at the start point, which is limited by the total number of nodes along the path excluding the source. Next, constraint (8i) removes the self-loops of all nodes (e.g., the self-loop of task #1 in Figure 3b). Constraint (8j) indicates that every edge on the path consumes one unit of the flow, which excludes the loops involving more than one node (e.g., the loop with tasks #2 and #3 in Figure 3b). Finally, constraints (8k,l) limit the range of $q_{j,k}$ and $r_{j,k}$, respectively.

Next, we analyze the winner selection problem for the incentive mechanism and have the following conclusion on its computational hardness.

**Theorem** **2.** *The winner selection problem in Equation (6) is NP-hard.*

**Proof.**  If we consider the cost of workers in Equation (6b) as the weight of items and the budget *B* as the total weight ω in the knapsack problem, the winner selection problem (6) is an instance of the knapsack problem. The only difference between the winner selection problem and the knapsack problem is the way to calculate the total value. In the knapsack problem, the total value is calculated by linearly adding up all the values of the selected items, while the total value of the winner selection problem is determined by adding up the cumulative values of paths according to Equation (5). Actually, the winner selection problem (6) is still a combinatorial optimization problem as a generalized knapsack problem, which is NP-hard. The remaining of the proof is similar to that for Theorem 1, which is skipped here for conciseness. □

## 4. Solutions to Formulated Problems

In this section, we propose a heuristic bidirectional searching algorithm for the path planning problem formulated in Equation (1) and an incentive mechanism, which first solves the winner selection problem in Equation (6) and then determines the payments based on the result of winner selection. At last, we prove that the proposed incentive mechanism is computationally efficient, individually rational, and truthful.

### 4.1. Heuristic Bidirectional Searching Algorithm

As analyzed in Section 3.4, the path planning problem is NP-hard. The search space of the solutions to the path planning problem is huge, since MCS is a large-scale sensing paradigm with massive tasks and workers. In addition, the workers need to consider not only the selection of tasks but also the order of the selected tasks, which affects the length of the designed path. Thus, it is hard to find an efficient algorithm to solve the path planning problem.

We propose a novel heuristic bidirectional searching algorithm to solve the path planning problem, given in Algorithm 1. As seen, there are three phases of Algorithm 1, i.e., forward searching, backward searching, and selection. In the forward searching, all the variables are first initialized (lines 1–5). Next, the task with the largest expected value is added into the path and all variables are updated (lines 6–12). The expected value of a task is discussed later. Here, $\hat{distance}$ in line 6 is different from distance in line 10. When we calculate the current travelling distance of the path, denoted by distance, we only need to consider the distance from the current location to the location of selected task, denoted by $d(location,loc_{t}\left[j\right])$. However, when we investigate whether a task is eligible to be selected, we have to consider two distances from the current location to the location of selected task and further to the destination. If the travelling distance limit is enough to take a specific task, while not enough to go to the destination after finishing this task, the path cannot include this task due to the travelling distance constraint. Thus, $\hat{distance}$ includes distances of two segments associated with a single task. Since distance does not count the last mile to the destination, the distance from the last task to the end point is added into the total travelling distance (line 13).

**Algorithm 1:** Heuristic bidirectional searching algorithm. **Input:** *T* (set of tasks), $w_{i}$ (worker $w_{i}$), $loc_{t}$ (locations of tasks), $loc_{s}$ (departure of worker $w_{i}$), $loc_{e}$ (destination of worker $w_{i}$), $l_{i}$ (maximum travelling distance of worker $w_{i}$), $\theta_{i}$ (maximum enegy cost of worker $w_{i}$), $\left\{g_{j}|\forall t_{j}\in T\right\}$ (energy costs of tasks), $\left\{v_{j}|\forall t_{j}\in T\right\}$ (original values of tasks) 
**Output:**
$F_{i}$ (travelling path of worker $w_{i}$)

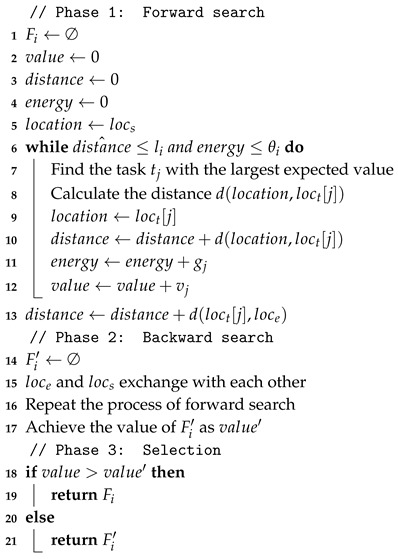



In the backward searching, we plan a path from the end point to the start point by the method mentioned above (lines 14–17). Since the distribution of tasks is not symmetrical in most instances, the path of backward searching is not the same as the path of forward searching, while the path of backward searching is also an optimized solution to the path planning problem (1). At last, we select a better solution as the final path from the results of forward searching and backward searching (lines 18–21).

In line 7, we select the task with the largest expected value, which is defined by

(9) $$\begin{array}{c} v\left(t_{j}\right)=v_{j}+E\left({\hat{l}}_{i},{\hat{\theta }}_{i}\right). \end{array}$$

Here, for every task $t_{j}\in T$, its expected value $v(t_{j})$ is divided into two parts, i.e., the current value and future value. The current value of task $t_{j}$ is its posted value $v_{j}$, while the future value is obtained by the function $E({\hat{l}}_{i},{\hat{\theta }}_{i})$ to evaluate the value that can be achieved in the future. If we just consider the current value $v_{j}$, the worker may first take the task with the largest current value, which is also subject to a very large energy cost or distance cost. In this case, the worker spends most resources on this task and has no space for other tasks, while there may exist a better solution of taking two tasks with the second and third largest values. Thus, we need a way to evaluate the value that is achievable with the remaining resources (i.e., the remaining energy limit ${\hat{\theta }}_{i}$ and remaining travelling distance limit ${\hat{l}}_{i}$) after taking the task with the largest value. Here, in the set of unvisited tasks, we simply take the task with the largest value that meets the requirements for ${\hat{l}}_{i}$ and ${\hat{\theta }}_{i}$. Note that task $t_{j}$ is already visited when we evaluate the future value and thus is not counted in this step. In this way, we obtain the future value $E({\hat{l}}_{i},{\hat{\theta }}_{i})$ of task $t_{j}$, which is added to the posted value $v_{j}$ to evaluate the expected total value of task $t_{j}$ as in Equation (9).

In the worst case, line 7 for the calculation of future work takes time O(m) to explore all *m* tasks and find the task with the largest value in the set of unvisited tasks. Meanwhile, the selection of the task in each iteration of lines 6–12 is also O(m) since we have to consider every task in the worst case. Thus, the time complexity of forward searching in lines 1–13 is at most $O(m^{2})$. The backward searching in lines 14–17 has the same complexity as the forward searching and the selection in lines 18–21 is O(1). Therefore, the overall time complexity of Algorithm 1 is $O(m^{2})$. Since Algorithm 1 is proposed for only one worker and workers are independent decision makers, the total computing time for the path planning problem is at most $O(nm^{2})$.

### 4.2. Truthful Incentive Mechanism

Based on the result of path planning, every worker $w_{i}\in W$ submits the bid $\left(S_{i},b_{i}\right)$ to the platform. Algorithm 2 gives the details of the proposed incentive mechanism for the platform to select the winners and determine the payments. There are two phases of Algorithm 2, i.e., winner selection and payment determination. In the winner selection, Γ is the union of winners’ task samples and v(Γ) is a function to compute the value of the union Γ according to Equation (5). The platform keeps selecting the worker with the largest marginal contribution of value per cost in each iteration (lines 5–12), after the initialization (lines 1–4). Here, *C* is used to record the current total cost and the budget constraint is checked in each iteration before adding the task set of a winner into Γ (line 7).

In the payment determination, the mechanism runs a virtual auction to determine the payment for every winner (lines 14–28) and sets the payment to 0 for others (lines 29–30). For every winner (line 13), the virtual auction starts after the initialization (lines 15–18) and excludes the winner itself (line 21). Then, the virtual auction keeps selecting the worker with the largest marginal contribution of value per cost in each iteration (lines 19–27). Here, $K_{i}$ is an integer that counts the iterations of lines 19–27. Meanwhile, a temporary payment is calculated in each iteration taking the selected worker and the winner into account (line 23). The payment to the winner is determined as the largest temporary payment in all iterations during the virtual auction (line 28). At last, after all the virtual auctions are completed, the mechanism returns the selected winners and corresponding payments (line 31).

Our proposed incentive mechanism is computationally efficient, individually rational, and truthful. First, we need to prove the following lemma, which will be used in our proofs for the three properties.

**Lemma** **1.** *Given* Γ*, if X⊆Y, we have:*

(10) $$\begin{array}{c} v(\Gamma \cup X)\leq v(\Gamma \cup Y), \end{array}$$

*where the equivalent is obtained only if X=Y.*

**Algorithm 2:** Truthful incentive mechanism. 
**Input:**
$\left\{v_{j}|\forall t_{j}\in T\right\}$ (original values of tasks), $\left\{S_{i}|\forall w_{i}\in W\right\}$ (task sets in bids), $\left\{b_{i}|\forall w_{i}\in W\right\}$ (costs in bids), *B* (cost budget) 
**Output:**
$\left\{x_{i}|\forall w_{i}\in W\right\}$ (indications of winners), $\left\{p_{i}|\forall w_{i}\in W\right\}$ (payments to workers)

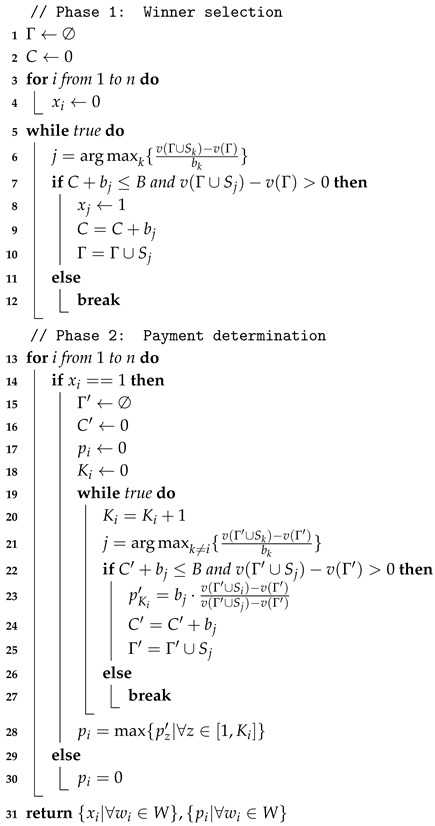

**Proof.**  According to Equation (5), an additional task sample brings extra value to the platform. When X⊂Y, i.e., Γ∪X⊂Γ∪Y, there are some additional task samples in Γ∪Y beyond those in Γ∪X. Thus, the value of Γ∪Y is larger than that of Γ∪X, i.e., v(Γ∪X)<v(Γ∪Y). When X=Y, i.e., Γ∪X=Γ∪Y, Γ∪Y and Γ∪X have the same data samples and thereby the same value. In summary, v(Γ∪X)≤v(Γ∪Y) and the equality is valid only if X=Y. □

In addition, the utilities of workers are defined as

(11) $$\begin{array}{c} u_{i}=\left\{\begin{array}{cc} p_{i}-c_{i}, & w_{i}\;\mathrm{wins},\\ 0, & \mathrm{otherwise}. \end{array}\right. \end{array}$$

**Theorem** **3.** *The proposed incentive mechanism in Algorithm 2 is computationally efficient.*

**Proof.**  The initialization of the winner selection (lines 1–4) takes a time of O(n) and the selecting process (lines 5–12) takes a time of at most O(n) when all the workers are selected as winners in the worst case. Thus, the time complexity of the winner selection phase is O(n). In the worst case, when all workers are winners, the mechanism runs a virtual auction for every worker. The time complexity of a single virtual auction (lines 15–28) is O(n) because the virtual auction is similar to the winner selection problem. There are *n* workers in total. Thus, the computing time of the payment determination (lines 13–30) is at most $O(n^{2})$. In total, the time complexity of the incentive mechanism in Algorithm 2 is $O(n^{2})$, which proves the computational efficiency. □

**Theorem** **4.** *The proposed incentive mechanism in Algorithm 2 is individually rational.*

**Proof.**  A mechanism is considered as individually rational when all workers receive non-negative utilities by bidding truthfully. On one hand, if a worker loses by bidding truthfully, the utility of the worker is zero according to Equation (11), which is non-negative. On the other hand, for every worker $w_{i}$ who wins by bidding truthfully, we assume that worker $w_{i}$ wins in the *z*-th iteration in the winner selection. It can be observed that, before the *z*-th iteration, Γ in the winner selection and $\Gamma^{\prime }$ in the virtual auction of worker $w_{i}$ are the same. In the *z*-th iteration of the virtual auction, worker $w_{j}$ is selected and the temporary payment $p_{z}^{\prime }=b_{j}\cdot \frac{V\left(\Gamma^{\prime }\cup F_{i}\right)-V\left(\Gamma^{\prime }\right)}{V\left(\Gamma^{\prime }\cup S_{j}\right)-V\left(\Gamma^{\prime }\right)}=b_{j}\cdot \frac{V\left(\Gamma \cup F_{i}\right)-V\left(\Gamma \right)}{V\left(\Gamma \cup S_{j}\right)-V\left(\Gamma \right)}$. In the winner selection, since worker $w_{i}$ wins in the *z*-th iteration and worker $w_{j}$ does not win before the *z*-th iteration, we have $\frac{V\left(\Gamma \cup F_{i}\right)-V\left(\Gamma \right)}{c_{i}}\geq \frac{V\left(\Gamma \cup S_{j}\right)-V\left(\Gamma \right)}{b_{j}}$. Applying this inequality into $p_{z}^{\prime }$, we can obtain $p_{z}^{\prime }\geq c_{i}$. Because the final payment is the maximum of all the temporary payments, we finally get $p_{i}\geq p_{z}^{\prime }\geq c_{i}$. According to Equation (11), the utility of worker $w_{i}$ as a winner is non-negative since $u_{i}=p_{i}-c_{i}\geq 0$. In conclusion, the utilities of all workers are non-negative regardless of whether they win or lose, which proves the individual rationality of the proposed mechanism. □

**Theorem** **5.** *The proposed incentive mechanism in Algorithm 2 is truthful.*

**Proof.**  A mechanism is considered as truthful when all workers receive the maximum utilities by bidding truthfully. In our proposed incentive mechanism, we need to prove that the workers are truthful with respect to both the task set and the cost. In the following proof, we consider any worker $w_{i}\in W$ and analyze three cases that worker $w_{i}$ may attempt to lie to improve its utility.

- **Case 1:**$S_{i}⊈F_{i}$. In this case, since worker $w_{i}$ includes tasks that it cannot complete in set $S_{i}$, the cost bid $b_{i}$ can be infinitely large according to Equation (3) if the platform detects and punishes such cheating behaviour. As shown in two sub-cases, worker $w_{i}$ loses anyway by bidding $\left(S_{i},b_{i}\right)$ and its utility cannot be improved regardless of whether it wins or loses by bidding truthfully.
  (i) When worker $w_{i}$ loses by bidding truthfully, its utility is 0, which is the same as that by bidding $S_{i}$.
  (ii) When worker $w_{i}$ wins by bidding truthfully, the utility is non-negative, which is reduced to 0 if it bids $S_{i}$.
- **Case 2:**$S_{i}\subseteq F_{i}$ and $b_{i}\geq c_{i}$.
  In this case, since $S_{i}\subseteq F_{i}$, we have $v\left(\Gamma \cup S_{i}\right)\leq v\left(\Gamma \cup F_{i}\right)$ according to Equation (10). Then, $v\left(\Gamma \cup S_{i}\right)-v\left(\Gamma \right)\leq v\left(\Gamma \cup F_{i}\right)-v\left(\Gamma \right)$, further $\frac{v\left(\Gamma \cup S_{i}\right)-v\left(\Gamma \right)}{b_{i}}\leq \frac{v\left(\Gamma \cup F_{i}\right)-v\left(\Gamma \right)}{b_{i}}\leq \frac{v\left(\Gamma \cup F_{i}\right)-v\left(\Gamma \right)}{c_{i}}$ because $b_{i}\geq c_{i}$. That is, worker $w_{i}$ cannot improve its marginal value contribution per cost by bidding $\left(S_{i},b_{i}\right)$. As a result, worker $w_{i}$ cannot change from lose to win by manipulation. Next, we consider three remaining sub-cases if worker $w_{i}$ lies and changes from lose to lose, from win to lose, and from win to win.
  (i) If worker $w_{i}$ loses by bidding truthfully and still loses by bidding $\left(S_{i},b_{i}\right)$, its utility is unchanged as 0.
  (ii) If worker $w_{i}$ wins by bidding truthfully, its utility is non-negative. If worker $w_{i}$ changes from win to lose by bidding $\left(S_{i},b_{i}\right)$, its utility reduces to 0 and is not improved by lying.
  (iii) If worker $w_{i}$ changes from win to win by bidding $\left(S_{i},b_{i}\right)$, according to Equation (11), its utility depends on its payment since the worker utility is always based on the unchanged true cost $c_{i}$. In its payment determination, since $S_{i}\subseteq F_{i}$, we have $v\left(\Gamma^{\prime }\cup S_{i}\right)-v\left(\Gamma^{\prime }\right)\leq v\left(\Gamma^{\prime }\cup F_{i}\right)-v\left(\Gamma^{\prime }\right)$, and further $b_{j}\cdot \frac{v\left(\Gamma^{\prime }\cup S_{i}\right)-v\left(\Gamma^{\prime }\right)}{v\left(\Gamma^{\prime }\cup S_{j}\right)-v\left(\Gamma^{\prime }\right)}\leq b_{j}\cdot \frac{v\left(\Gamma^{\prime }\cup F_{i}\right)-v\left(\Gamma^{\prime }\right)}{v\left(\Gamma^{\prime }\cup S_{j}\right)-v\left(\Gamma^{\prime }\right)}$. Thus, the temporary payment by biding $S_{i}$ is not larger than that by bidding $F_{i}$ in all steps. Therefore, the final payment by bidding $S_{i}$ is not larger than that by bidding $F_{i}$, and its utility is not improved by untruthful bidding $\left(S_{i},b_{i}\right)$.
- **Case 3:**$S_{i}\subseteq F_{i}$ and $b_{i}<c_{i}$. In this case, we consider four sub-cases depending on whether worker $w_{i}$ loses or wins when bidding truthfully and untruthfully.
  (i) If worker $w_{i}$ wins by bidding truthfully, its utility is non-negative. When worker $w_{i}$ changes from win to lose by bidding $\left(S_{i},b_{i}\right)$, its utility is decreased to 0.
  (ii) If worker $w_{i}$ changes from win to win by bidding $\left(S_{i},b_{i}\right)$, its utility cannot be improved as proved in Case 2, as the payment of worker $w_{i}$ does not depend on its cost bid $b_{i}$.
  (iii) If worker $w_{i}$ loses by bidding truthfully, its utility is 0. When worker $w_{i}$ changes from lose to lose by bidding $\left(S_{i},b_{i}\right)$, its utility is still 0.
  (iv) If worker $w_{i}$ changes from lose to win by bidding $\left(S_{i},b_{i}\right)$, we need to evaluate the change of its utility. Assume that the maximum temporary payment is obtained at the *z*-th step in its virtual auction. Then, we have $p_{i}=p_{z}^{\prime }=b_{j}\cdot \frac{v\left(\Gamma^{\prime }\cup S_{i}\right)-v\left(\Gamma^{\prime }\right)}{v\left(\Gamma^{\prime }\cup S_{j}\right)-v\left(\Gamma^{\prime }\right)}\leq b_{j}\cdot \frac{v\left(\Gamma^{\prime }\cup F_{i}\right)-v\left(\Gamma^{\prime }\right)}{v\left(\Gamma^{\prime }\cup S_{j}\right)-v\left(\Gamma^{\prime }\right)}$, since $v\left(\Gamma^{\prime }\cup S_{i}\right)\leq v\left(\Gamma^{\prime }\cup F_{i}\right)$ according to Equation (10). As we already know that worker $w_{i}$ loses by bidding $\left(F_{i},c_{i}\right)$ in the winner selection, we can obtain $\frac{v\left(\Gamma \cup F_{i}\right)-v\left(\Gamma \right)}{c_{i}}<\frac{v\left(\Gamma \cup S_{j}\right)-v\left(\Gamma \right)}{b_{j}}$ at the *z*-th step of the winner selection. We can rewrite it as $\frac{v\left(\Gamma^{\prime }\cup F_{i}\right)-v\left(\Gamma^{\prime }\right)}{c_{i}}<\frac{v\left(\Gamma^{\prime }\cup S_{j}\right)-v\left(\Gamma^{\prime }\right)}{b_{j}}$ because Γ and $\Gamma^{\prime }$ are the same before the *z*-th step. Thus, we have $p_{i}\leq b_{j}\cdot \frac{v\left(\Gamma^{\prime }\cup F_{i}\right)-v\left(\Gamma^{\prime }\right)}{v\left(\Gamma^{\prime }\cup S_{j}\right)-v\left(\Gamma^{\prime }\right)}<c_{i}$. Therefore, $u_{i}=p_{i}-c_{i}<0$. As seen, the utility of worker $w_{i}$ is reduced to be negative by bidding $\left(S_{i},b_{i}\right)$.

Based on the above analysis, we can see that every worker maximizes its utility only by bidding truthfully in the data set and cost. Therefore, the proposed mechanism is truthful. □

## 5. Numerical Results and Discussion

In this section, we evaluate the performance of the proposed heuristic bidirectional searching algorithm and incentive mechanism by comparing them with the baselines. For the path planning problem, we consider two baseline algorithms and reformulate the path planning problem to obtain the optimal solution. For the incentive mechanism design, we leverage the well-known VCG mechanism to achieve the same performance as the proposed incentive mechanism, and then compare their payments for such performance.

### 5.1. Baselines for Path Planning

As proved in Theorem 1, the path planning problem is NP-hard. Based on the ILP formulation in Equation (8) for the problem, we can obtain the optimal solution using some ILP solvers when the problem size is not large. In addition, we consider some heuristic approximation algorithms, which are needed for large-sized problems. Since the objective in the path planning problem (1) is the largest value of the path, it is intuitive to keep selecting the task with the largest value until the constraints are not satisfied. This method, known as *the value-first algorithm* in the following, is simple and effective. However, the drawback is that it is short-sighted and never considers the remaining resources. As a result, it may not achieve a large final value.

From another perspective, we can keep taking the task with the smallest cost for resources to find a feasible path. Here, there are two resources in the path planning problem, i.e., energy cost and travelling distance. In [40], the authors proposed an algorithm called minimum weighted sum first heuristic, which gives weights to two different constraints. Following this idea, we define the cost of resources of each worker $w_{i}$ in a similar way as $\frac{d_{j}}{{\hat{l}}_{i}}+\frac{e_{j}}{{\hat{\theta }}_{i}}$. Here, $d_{j}$ is the travelling distance cost to finish task $t_{j}$ and ${\hat{l}}_{i}$ is the remaining travelling distance limit of worker $w_{i}$. In the second term, $e_{j}$ is the energy cost to finish task $t_{j}$ and ${\hat{\theta }}_{i}$ is the remaining energy limit of worker $w_{i}$. Applying this resource cost definition to the path planning problem, we consider *the resource-first algorithm*, which keeps selecting the task with the smallest resource cost as long as the constraints are satisfied.

### 5.2. VCG Mechanism

In order to evaluate the performance of our proposed incentive mechanism, we use the well-known VCG mechanism as a benchmark. However, we cannot directly use the winner selection problem (6) in the VCG mechanism because problem (6) involves both the values of the platform toward tasks and the costs of workers in the bids. Thus, we have to adapt the winner selection problem for the VCG mechanism. Let Λ denote the maximum value in Equation (6a) obtained by the proposed mechanism. Then, we define the winner selection problem in the VCG mechanism as

(12a) $$\begin{array}{c} \min.\;\;\;\sum_{w_{i}\in W}x_{i}\cdot b\left(S_{i}\right), \end{array}$$

(12b) $$\begin{array}{c} s.t.\;\;\;\;\;\sum_{t_{j}\in T}{\hat{v}}_{j}\geq \Lambda , \end{array}$$

(12c) $$\begin{array}{c} \;\;\;\;\;\;\;\;x_{i}=\left\{0,1\right\},\forall w_{i}\in W. \end{array}$$

Here, the VCG mechanism can determine the winners such that the achieved value is at least Λ as constrained by Equation (12b). To calculate the payments for these selected winners, there remains a problem before the VCG mechanism can be applied. If the path of one specific worker $w_{i}$ has a very large value, worker $w_{i}$ would be selected as a winner. When determining the payment for winner $w_{i}$, the VCG mechanism needs to exclude $w_{i}$ from problem (12) to compute the total value. The VCG payment is the difference between the total value without worker $w_{i}$’s presence and the total value when worker $w_{i}$ participates, but its contribution to the total value is excluded. Hence, it is possible that the maximum achievable value without this worker is smaller than Λ, which means problem (12) does not have a feasible solution without worker $w_{i}$. The VCG payment to worker $w_{i}$ becomes infinity. In order to solve this issue, we consider a dummy worker with a task set of value Λ and a cost bid β. As a result, the payment to every winner is not more than β, which serves as the reserve wage for the platform to recruit a worker.

In a small-scale scenario, the modified winner selection problem (12) for the VCG mechanism can be solved by a brute-force algorithm to obtain the optimal solution. It is worth mentioning that we can also obtain the optimal solution to the original winner selection problem (6) in our proposed incentive mechanism by exhaustive search.

### 5.3. Simulation Settings

**Figure 4:**
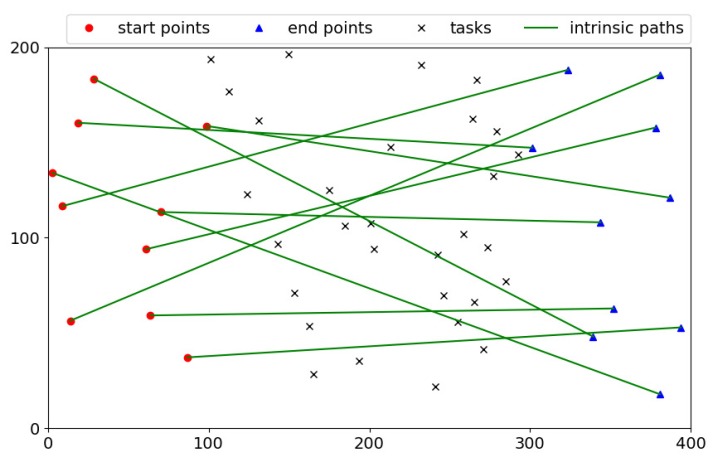
Simulation scenario.

After giving the baselines, we conduct comprehensive simulations to evaluate the performance of our proposed heuristic bidirectional searching algorithm and incentive mechanism. Table 2 lists the values of the key simulation parameters. First of all, we set up the sensing region as a 400 × 200 rectangle. The start points and end points of workers, and positions of tasks are generated uniformly within this area, respectively. Figure 4 shows these locations and the intrinsic paths of workers. As seen, the start points of workers are located in the residential area (1≤x≤100) and their end points fall into the business area (300≤x≤400). The sensing tasks are distributed in the middle square area (100≤x≤300).

There are 30 tasks and 10 workers in the scenario. For tasks, the energy costs are randomly selected from 1 to 3 as an integer number indicating the low level, mediate level, and high level of energy consumption. The original values of tasks are randomly selected in the range of 5 to 10 as an integer number. For workers, the maximum travelling distances are 1.2 times of the length of their intrinsic paths. The energy limits of workers are all set to 30. In the simulation for the incentive mechanisms, the true costs per distance of worker are the same as 1 and the cost budget of the platform is 500.

### 5.4. Result of Path Planning

**Figure 5:**
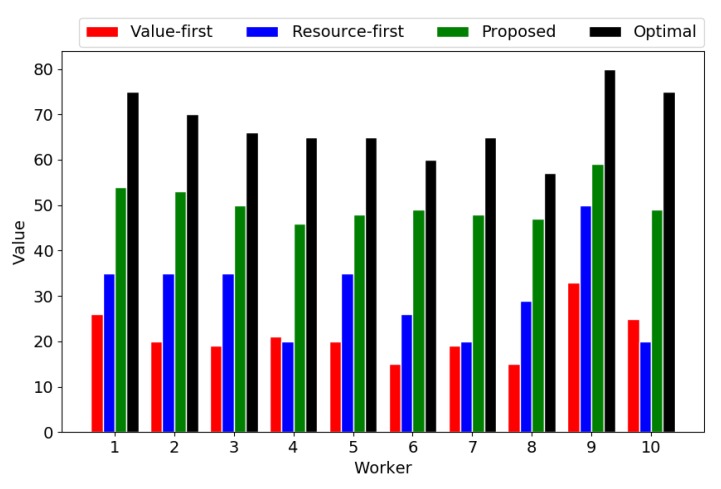
Values of the planned paths.

The most important performance metric for the path planning problem is the objective value, i.e., the value of the path. Figure 5 shows the values of all paths with different algorithms. We can see that the proposed heuristic bidirectional searching algorithm outperforms two baselines in all paths. Simultaneously, the results of the proposed algorithm are very close to the optimal values in some paths.

**Figure 6:**
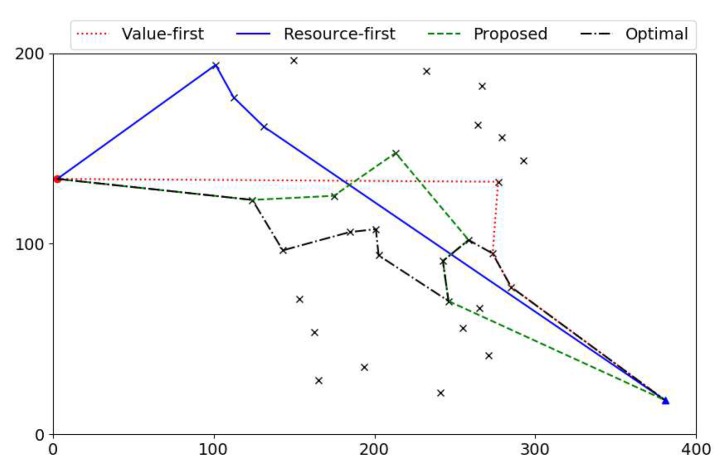
Paths of different algorithms.

In order to show the details of the paths obtained by different algorithms, we take worker #10 as an example. Figure 6 shows four paths of worker #10 designed by different algorithms. It is obvious that the optimal solution has the longest path and the largest value (as has been shown in Figure 5). The proposed algorithm takes the same first task as the optimal solution, then goes to another direction and achieves a moderate value about two-thirds that of the optimal solution. The resource-first algorithm takes the first task with the smallest cost of resources, while this leads the worker to an isolated area with few tasks. As a consequence, the resource-first algorithm finishes the minimal number of tasks and obtains the worst value. The value-first algorithm selects the first task with the largest value, which is far away from the start point. In this case, the worker misses some tasks near the start point, which may potentially increase the value of the path. Eventually, the value-first algorithm receives a value less than half of that of the optimal solution.

**Figure 7:**
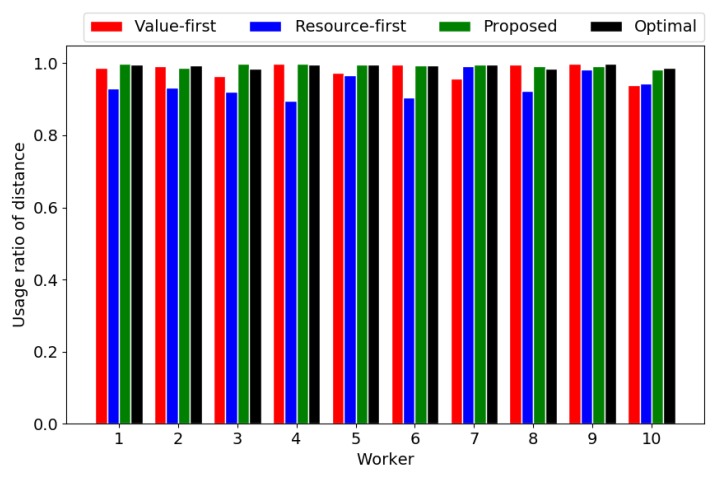
Usage ratio of travelling distance.

**Figure 8:**
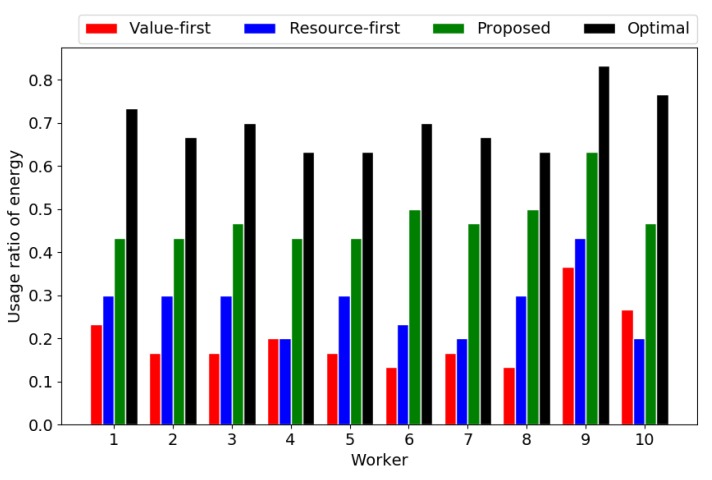
Usage ratio of energy.

Figure 7 and Figure 8 show the usage ratios of travelling distance and energy. It can be seen that the maximum travelling distance is the main constraint of paths, whose usage ratio approaches 1 in all paths and all four of the algorithms. On the other hand, the usage of the energy in the optimal solution is the largest, followed by the proposed algorithm. This implies that the proposed algorithm has a better balance in the usage of different resources (i.e., travelling distance and energy) than the baselines, although slightly worse than the optimal solution. The proposed algorithm outperforms the baselines mainly because it can utilize the resources in a balanced manner.

### 5.5. Result of Incentive Mechanism

Based on the paths designed by the proposed algorithm, all workers submit their bids to the platform. Then, the incentive mechanism first selects the winners. Table 3 lists the selection of winners in the proposed mechanism, VCG mechanism, and the optimal solution to the winner selection problem (6). As seen, the proposed mechanism and VCG mechanism have the same set of winners, which makes the comparison of payments discussed later meaningful. The optimal solution has the same number of winners but different from those selected by the proposed mechanism. The values of all completed tasks in the proposed mechanism and VCG mechanism are the same as 269.57 because of the same winner selection, while the value of the optimal solution is 278.21. As seen, our proposed mechanism is efficient in winner selection approaching the performance of the optimal solution. In addition, the used costs of the proposed mechanism, VCG mechanism, and optimal solution are 468.42, 468.42, and 485.50, respectively. All the costs are smaller than the cost budget 500.

**Figure 9:**
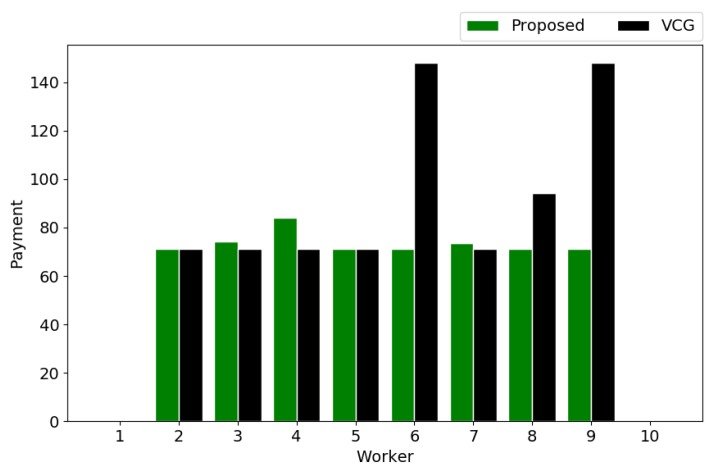
Payments to winners.

The payments to the winners are determined after the winner selection. Figure 9 shows the payments to winners in both the proposed mechanism and VCG mechanism. As seen, most winners have the almost same payments in these two mechanisms, while some winners are paid significantly larger in the VCG mechanism than in the proposed mechanism, e.g., workers #6 and #9. As a result, in our scenario, the total payment in the VCG mechanism (746.23) is larger than that in the proposed mechanism (587.78). It is worth noting that the payment rules in the proposed mechanism and the VCG mechanism are totally different. Therefore, the relationship of the total payments in both mechanisms may vary in other scenarios. However, when the distribution of tasks is dense and the values of tasks are close, the total payments in both mechanisms are expected to be close.

**Figure 10:**
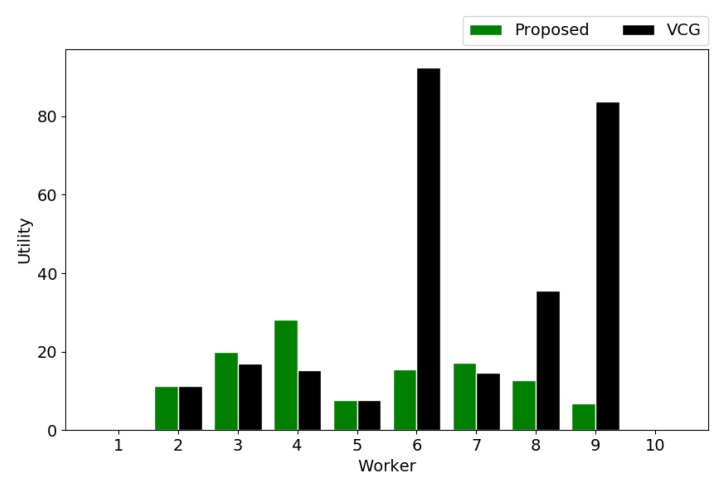
Utilities of workers.

According to the true costs of workers and the payments to winners, we obtain the utilities of workers, shown in Figure 10. It is noticed that the utilities of losers are 0. As seen, the utilities are similar for most workers in both mechanisms. However, due to the non-frugal payments in the VCG mechanism, some winning workers achieve utilities that are significantly higher than in the proposed mechanism.

**Figure 11:**
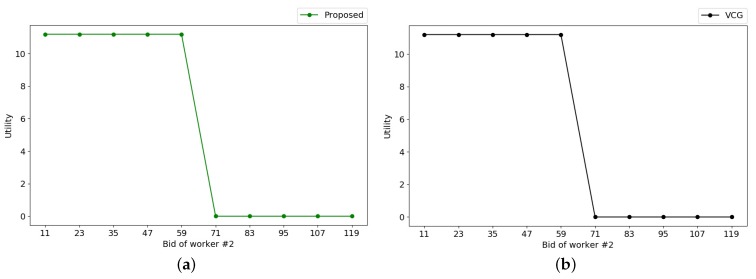
Truthfulness of both mechanisms. (**a**) proposed mechanism; (**b**) VCG mechanism.

It is known that the most critical property of the incentive mechanism is truthfulness. In order to show the truthfulness both in the proposed mechanism and VCG mechanism, we take worker #2 who is a winner as an example. We change the cost bid of worker #2 from 0 to two times of its true cost. Figure 11 shows how the utility of worker #2 varies with its cost bid. As seen in Figure 11a with the proposed mechanism, worker #2 achieves the highest utility when it bids truthfully. Even if worker #2 bids a cost lower than its true cost, its utility is the same and will not be improved. A similar observation can be seen in Figure 11b with a VCG mechanism. The results in Figure 11 verify the truthfulness of both mechanisms.

## 6. Conclusions

In this paper, we built a technical framework for MCS with novel solutions to two key problems, i.e., path planning and incentive mechanism design. To take into account user mobility while protecting workers’ privacy, we studied the path planning problem in the worker-centric mode so that the workers plan their own paths. The heuristic bidirectional searching algorithm addresses the computational complexity of the path planning problem to obtain an efficient solution. After the workers decide their bids according to their paths, the platform runs an auction-based incentive mechanism to determine the task allocation and corresponding payments to the winning workers. The proposed incentive mechanism satisfy three desirable properties, especially truthfulness with respect to both the task sets and costs of workers. The simulation results validate the high efficiency and good properties of the proposed solutions, and show performance improvement over the benchmarks in terms of total task value, workers’ utilities and payments.

As already known, one drawback of the worker-centric mode is that the workers may have strong competition when they are interested in the same tasks. In this case, the platform-centric mode is a better option since the platform can coordinate all the workers in this mode. However, some workers as independent decision makers may not be willing to have the platform control their paths. Therefore, we are planning to develop a hybrid method to address this issue in the future. For example, the workers can bid for the tasks as a group. Then, the key challenge is to create stable groups of workers and further assign the tasks within each winning group. In addition, it would be interesting to implement the proposed framework in a real MCS application and carry out small-scale tests. A mobile client can be built upon the Android system to plan paths and determine bids, while a Web-based backend server can post tasks, collect bids, and run an auction to allocate tasks.

## Figures and Tables

**Table 1:** Notation definitions.

Notations	Definitions	Notations	Definitions
*T*	Set of tasks	$F_{i}$	Path and true data set of worker *i*
$t_{j}$	Task *j*	*B*	Budget of total cost
*W*	Set of workers	$S_{i}$	Data set in the bid of worker *i*
$w_{i}$	Worker *i*	$b_{i}$	Cost in the bid of worker *i*
$v_{j}$	Original value of task *j*	$c_{i}$	True cost of worker *i*
$g_{j}$	Energy cost of task *j*	$\gamma_{i}$	True cost per distance of worker *i*
$h_{i}$	Intrinsic path’s length of worker *i*	$x_{i}$	Winner assignment of worker *i*
$\theta_{i}$	Energy limit of worker *i*	$y_{j}$	Number of competitors for task *j*
$l_{i}$	Maximum travelling distance of worker *i*	${\hat{v}}_{j}$	Cumulative value of task *j*

**Table 2:** Simulation parameters.

Parameter	Value
Number of tasks *m*	30
Number of workers *n*	10
Energy cost of tasks $g_{j}$	1∼3
Original value of tasks $v_{j}$	5∼10
Maximum travelling distance of workers $l_{i}$	$1.2\times h_{i}$
Energy limit of workers $\theta_{i}$	30
True cost per distance of workers $\gamma_{i}$	1
Cost budget of the platform *B*	500

**Table 3:** Results of winner selection.

Mechanism	Winner Selection
Proposed mechanism	{0,1,1,1,1,1,1,1,1,0}
VCG mechanism	{0,1,1,1,1,1,1,1,1,0}
Optimal solution	{0,1,0,1,1,1,1,1,1,1}
